# Successful management of severe generalized tetanus in a 23‐year man with phenobarbital adjuvant: A case report

**DOI:** 10.1002/ccr3.8286

**Published:** 2023-12-15

**Authors:** Arezoo Ahmadi, Elahe Karimpour‐Razkenari, Ramin Ansari, Mahforouzalsadat Marashi, Hamidreza Sharifnia, Atabak Najafi, Mojtaba Mojtahedzadeh

**Affiliations:** ^1^ Department of Anesthesiology and Critical Care, School of Medicine, Sina Hospital Tehran University of Medical Sciences Tehran Iran; ^2^ Department of Clinical Pharmacy, Faculty of Pharmacy Tehran University of Medical Sciences Tehran Iran; ^3^ Department of Infectious Disease and Tropical Medicine, School of Medicine, Sina Hospital Tehran University of Medical Sciences Tehran Iran

**Keywords:** intensive care unit, phenobarbital, refractory spasm, tetanus

## Abstract

Generalized tetanus is still a global concern with a mortality rate of up to 50%, especially in low and middle‐income countries. We reported a 23‐year‐old man from Afghanistan admitted to emergency department, with the chief complaint of generalized severe spasms and lockjaw. The patient had skin lesions and had never been vaccinated against tetanus. He intubated and admitted to the intensive care unit (ICU) with diagnose of severe generalized tetanus. After receiving tetanus immunoglobulin and intravenous metronidazole, a combination therapy of midazolam, propofol, atracurium, and morphine was administered. Due to the refractory muscular spasms intravenous phenobarbital started and little by little recovery was achieved. The patient receiving the first two doses of the Td vaccine, and discharged on Day 42 of hospitalization with no symptom recurrence. This case management showed adding phenobarbital to severe tetanus treatment regimen could significantly reduce refractory spasm caused by tetanus, also decrease other medication requirement.

## Graphical abstract

The process of successful management of severe generalized tetanus.

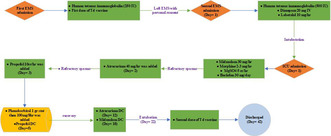

## 1 INTRODUCTION

Tetanus is caused by Clostridium tetani with a mortality rate of 4.2%–50%, even though it is a vaccine‐preventable disease.^1^ Higher mortality rates have been reported from centers with limited intensive care and ventilator support.^2^ Despite the dramatic reduction in the prevalence of the disease thanks to vaccination programs, tetanus remains a global problem, particularly in low and middle‐income countries due to lack of vaccination or not receiving the booster dose.^3^, ^4^

As a toxin‐producing, anaerobic gram‐positive spore‐forming bacterium, *Clostridium tetani* produces tetanolysin and tetanospasmin. Besides the role of tetanolysin in intensifying wound damage and providing anaerobic condition for bacterium growth, most of the clinical manifestations of tetanus result from tetanospasmin, which inhibits gamma amino butyric acid (GABA)‐ergic and glycinergic neurons.^5^, ^6^

The diagnosis of generalized tetanus is based on the history of the injury and clinical features.^7^ Attributable to the limited capacity for clinical trials and available management options, there are still limitations in evidence‐based management strategies for the disease.^8^ Nevertheless, admission to intensive care unit (ICU) is offered for patients with high‐risk tetanus, and treatments such as early tracheostomy and administering benzodiazepines, magnesium sulfate, and morphine are effective and recommended as first‐line therapy alongside supportive care.^4^ Phenobarbital is a barbituric acid derivative that acts as a non‐selective central nervous system inhibitor by mimicking the action of GABA in the brain. It enhances the effects of GABA by facilitating the passage of chlorine ions (Cl) through Cl channels in GABA receptors. Therefore, it appears that phenobarbital may be able to reverse the inhibitory effects of tetanospasmin on the GABA receptor.^9^, ^10^

In this paper, we introduce a case of severe generalized tetanus with respiratory failure, whose spasm did not improve with full doses of benzodiazepines and muscle relaxant agents, nevertheless he was finally recovered with phenobarbital.

## 2 CASE REPORT

A 23‐year‐old man (weight = 70 kg), construction worker from Afghanistan with lockjaw leading to dysphagia and intermittent muscular spasm in the upper and lower limbs was admitted to the emergency department of educational hospital, affiliated with Tehran University of Medical Sciences.He has no notable medical history, but a nail stuck into his right sole, 3 days before admission.The patient was not vaccinated against tetanus. On admission day, he had stable hemodynamics (blood pressure (BP) = 121/79 mmHg, heart rate (HR) = 90 beat/min, respiratory rate (RR) = 21 breath/min, SPO2 = 98%, Temperature = 37.5°C) and normal laboratory results (complete blood count (CBC), erythrocyte sedimentation rate (ESR), C‐reactive protein (CRP), liver function test (LFT), blood urea nitrogen (BUN), creatinine (Cr), arterial blood gas (ABG), and serum electrolytes). He received human tetanus immunoglobulin (250 IU) and the first dose of the tetanus‐diphtheria toxoid (Td) vaccine (0.5 mL) intramuscularly, but he left the emergency department with personal consent. Unfortunately, 3 days later he was readmitted with loss of consciousness (Glasgow coma scale = 10), severe generalized spasm and rigidity in the lower and upper limbs. Other clinical manifestations included sustained spasms of the facial muscles (Risus sardonicus), severe contractions of masseter muscles (lockjaw), and sweating. His hemodynamics were unstable (BP = 178/90 mmHg, HR = 180 beat/min, RR = 45 breath/min, SPO2 = 85%, Temperature = 38.5°C). According to the Ablett classification of tetanus severity,^11^ the patient categorized as very severe. After wound cleaning and debridement, he received the second dose of tetanus immune globulin (500 IU), diazepam (20 mg), and labetalol 10 mg/h in the emergency department. Meanwhile he intubated and admitted to the ICU. His laboratory results upon admission to the ICU given in Table 1, indicating elevated levels of ESR, CRP, Lactate dehydrogenase (LDH), and Creatine kinase (CPK), and diminished serum calcium (Ca corrected = 7.6), however blood and urine culture were negative.

**TABLE 1:** Patient's laboratory data at the admission and discharge from ICU.

Parameter	ICU admission	Discharge from ICU	Normal value^a^
WBC (cell/mm^3^)	13,100	6200	4100–10,100
Hgb (g/dL)	11	11.2	12–16
MCV (fl)	88.9	89.6	77–94
RBC (million/mm^3^)	3.58	4.10	4.2–5.8
Platelet (cell/mm^3^)	322,000	316,000	150,000–400,000
ALT (U/L)	24	18	<41
AST (U/L)	36	28	<37
Bilirubin total (mg/dL)	0.47	0.62	0.1–1.2
Bilirubin Direct (mg/dL)	0.17	0.22	<0.3
ALP (U/L)	426	126	70–306
ESR (mmol/h)	84	10	Male < 22
CRP (mg/L)	74	6	Adult < 6
LDH (U/L)	2518	120	350<
CPK (U/L)	10,852	186	Male < 174
PT (s)	16.3	16.3	11–15
INR	1.3	1.2	1–1.2
APTT (s)	38.2	36	25–40
Serum Cr (mg/dL)	0.92	1.02	0.7–1.4
Urea (mg/dL)	24	32	15–50
Na (meq/L)	139	137	135–145
K (meq/L)	3.78	4.2	3.5–5
Mg (mg/dL)	3	2.4	1.6–2.6
Ca (mg/dL)	6	7.8	8.6–10.2
P (mg/dL)	2.7	2.2	2.5–5
Alb (g/dL)	2	2.8	3.5–5.5

^a^ Normal range in our hospital's laboratory.

The patient was isolated in a dark, quiet and isolated room in the ICU because any sensory stimuli—including light, touch, and loud noise—would trigger the spasms. Despite receiving midazolam (30 mg/h), morphine (2–3 mg/h), and MgSO_4_ (5 cc/h) infusion, the patient experienced severe and refractory spasms permanently. As a consequence of the insufficiency of these medications for spasms controlling, a neuromuscular blocking agent, Atracurium infusion (40 mg/h), was added to other medication as well. But the spasms were remained refractory to all these medications, so Propofol infusion (10 cc/h) was started on the third day of ICU admission, then the spasms subsided comprehensibly. Owing to the concern of the propofol infusion syndrome probability and similar spasm inhibition mechanism, the therapeutic regimen was followed by intravenous phenobarbital (1 gr as loading dose and 100 mg/8 h as maintenance dose) after 48 h of propofol starting. The spasm gradually declined after administering phenobarbital; therefore on Day 10 of ICU admission, atracurium and midazolam were gently tapered down (they were discontinued on Day 12 and 18 of ICU admission, respectively). Finally, he was extubated from mechanical ventilation in Day 22 of ICU admission. It is worth mentioning that the phenobarbital serum level was at the therapeutic range (between 25 and 30 mg/L, therapeutic level is 10–40 mg/L in our hospital laboratory) during the survey. Furthermore, the addition of phenobarbital to the treatment regimen did not cause any adverse effect that require treatment such as hypotension, bradycardia, dermatologic reaction, agranulocytosis, and thrombocytopenia.

It should be noted that because of MgSO_4_ infusion and the patient's low calcium level at baseline, we have to administered calcium gluconate supplement intravenously to keep his corrected calcium level above 8.5 mg/dL. Meanwhile, the patient received pantoprazole and heparin for stress ulcer and DVT prophylaxis as well as intravenous metronidazole and oral baclofen from the first day, Furthermore tracheostomy was performed on the sixth day of ICU admission. On the other hand, the patient need high calorie nutrition due to continuous muscle over activity and spasms, since he could not tolerate enteral nutrition and had excessive residue volume, total parenteral nutrition (TPN) started on Day 4 of ICU admission. Finally, the ESR, CRP, LDH, and CPK levels decreased gradually and normalized on Day 28 as shown in Table 1, and he was discharged with no symptom recurrence on Day 42 of hospitalization, after receiving the second dose of the Td vaccine. A written informed consent was obtained from the patient in order to publish this case report, and it was approved by the Research Ethics Committee of our Hospital.

## 3 DISCUSSION

Tetanus is still a global concern since the World Health Organization reported 14,751 tetanus cases in 2019.^8^ Generalized tetanus was the most common type, accounting for about 80% of tetanus patients.^12^ ICU admission has been suggested for generalized tetanus for better monitoring, as well as timely diagnosis of complications that will reduced morbidity and mortality rates.^5^, ^13^ Although there are some protocols for tetanus management, there is a lack of substantial evidence for tetanus management strategies.^1^, ^13^ Hence, the management of tetanus is challenging even for the most experienced physicians.

In this case, although we administered tetanus immune globulin (500 IU) and intravenous metronidazole, midazolam, morphine, MgSO_4_ on the first day of ICU admission, the spasms continued, and be worsen over the first 3 days. This might be due to the toxin that had already entered the motor neurons and was progressing toward the central nervous system.^6^ It has been reported that tetanospasmin inhibits the action of enkephalins, which may play a role in modulating the autonomic nervous system.^14^ Therefore the propofol adding to first‐line medication might held to controlling this refractory spasms. Although no preferred combination therapy is available thus far, some studies suggest the addition of propofol, neuromuscular blockers, or a combination of both when there was no adequate clinical response to benzodiazepines.^7^, ^15^ So, atracurium and then propofol were added because of the patient's resistant spasm and high sensitivity to any sensory stimulus.

Since the patient's spasms began to decrease after propofol administration, and also due to the concern about the probability of propofol infusion syndrome, we decided to change it to intravenous phenobarbital, with regards to similarity in the antispasmodic mechanism of these agents. There are some case reports supporting the use of phenobarbital in generalized and neonatal tetanus, although some of them have shown no mortality benefit.^16^ A meta‐analysis of studies on children with tetanus reported diazepam alone is more beneficial on controlling tenues and reducing mortality than if it is combined with phenobarbital (RR of death 0.36; 95% CI 0.15–0.86; risk difference 12.22; 95% CI −0.38 to −0.06).^17^ However, the combination of diazepam and phenobarbital compered to diazepam alone has demonstrated a significantly shorter clinical course and hospitalization.^18^ This evidence confirming the efficacy and safety of phenobarbital in tetanus management, which suggests adding phenobarbital to primary treatment of severe tetanus could be a favorable choice.

There are several reports about the role of MgSO_4_ in patients with tetanus. It might be used to resolve muscle spasms and autonomic instability (including hypertension and tachycardia) and reduce the need for benzodiazepines and neuromuscular blockers. On the other hand, some studies showed that MgSO_4_ has no significant effect on mortality and should not be used as monotherapy in these patients.^19^ Beyond that intrathecal baclofen has been successfully used in patients with spasms that are resistant to neuromuscular blocking agents. It seems that intrathecal baclofen could shorten the duration of mechanical ventilation and reduce the rate of mortality,^20^ but intrathecal baclofen was not available in our setting, so we decided to administer oral baclofen (30 mg per day). However, the therapeutic response to these medication was insufficient in our patient.

On Day 10 of ICU admission and after phenobarbital starting, the patient showed a significant reduction in clinical manifestations, although intermittent muscle spasms continued until Day 32. On the other hand, due to the patient's severe pain and spasms and to follow the ethical considerations, we decided to add medications as soon as possible in the failure of a complete response to first‐line therapy. Due to the use of combination therapy to manage severe muscle over activity in this case, it is not clear which drug yielded the exact clinical benefit but it seems that adding phenobarbital help effectively in controlling the symptoms, sever spasm and tapering off other medication especially midazolam infusion, with no additional adverse effect.

Since tetanus infection does not provide natural immunity, patients need a full course of vaccination. Our patient received one dose of Td vaccination on his first admission and the second dose during discharge. The next dose should be administered 6–12 months later. Although it has been suggested that vaccine‐naïve patients should receive at least one dose of diphtheria‐tetanus‐acellular pertussis (DTaP) vaccination,^21^ due to the unavailability of DTaP in our region we considered Td for all three doses.

## 4 CONCLUSION

Although there are some guidelines for managing severe generalized tetanus, no preferred combination therapy has been established until now. Our study found adding phenobarbital to first‐line medicine could subside sever spasm more quickly with no adverse reaction. However, further studies are required to understand the best combination and dosage of medications for severe tetanus management.

## AUTHOR CONTRIBUTIONS

**Arezoo Ahmadi:** Conceptualization; investigation; methodology; project administration; supervision; writing – review and editing. **Elahe Karimpour‐Razkenari:** Data curation; writing – original draft; writing – review and editing. **Ramin Ansari:** Writing – original draft. **Mahforouzalsadat Marashi:** Investigation. **Hamidreza Sharifnia:** Investigation; project administration. **Atabak Najafi:** Supervision. **Mojtaba Mojtahedzadeh:** Supervision.

## FUNDING INFORMATION

The authors received no financial support for research, authorship, or publication of the paper.

## CONFLICT OF INTEREST STATEMENT

On behalf of all of the authors, the corresponding author declares no conflict of interests.

## ETHICS STATEMENT AND CONSENT TO PARTICIPATE

Written informed consent for publication of their clinical details and/or clinical images was obtained from the patient/parent/guardian/relative of the patient. A copy of the consent form is available for review by the Editor of this journal.

## Data Availability

The data that support the findings of this study are available on request from the corresponding author. The data are not publicly available due to privacy or ethical restrictions.

## References

[1] Shah B . A review on pharmacological management of generalised tetanus. Gen Med. 2018;06(1):2‐6.

[2] Attygalle D , Rodrigo N . New trends in the management of tetanus. Expert Rev Anti Infect Ther. 2004;2(1):73‐84. 15482173 10.1586/14787210.2.1.73

[3] Hasnain MG , Maruf S , Nath P , et al. Managing severe tetanus without ventilation support in a resource‐limited setting in Bangladesh. Am J Trop Med Hyg. 2018;99(5):1234‐1238. 30203739 10.4269/ajtmh.18-0180 PMC6221245

[4] Govindaraj GM , Riyaz A . Current practice in the management of tetanus. Crit Care. 2014;18:145. 25042788 10.1186/cc13894 PMC4057478

[5] Lisboa T , Ho Y‐L , Henriques Filho GT , et al. Guidelines for the management of accidental tetanus in adult patients. Rev Bras Ter Intensiva. 2011;23:394‐409. 23949453

[6] George EK , De Jesus O , Vivekanandan R . *Clostridium tetani* Infection. StatPearls. StatPearls Publishing; 2021. 29494091

[7] Seo SW , Lee J , Yoo B‐G , Kim J , Huh S‐Y . Autonomic instability in severe tetanus: a case report. Ann Clin Neurophysiol. 2021;23(2):117‐120.

[8] Karnad DR , Gupta V . Intensive care management of severe tetanus. Indian J Crit Care Med. 2021;25(Suppl 2):S155‐S160. 34345131 10.5005/jp-journals-10071-23829 PMC8327798

[9] Twyman RE , Rogers CJ , Macdonald RL . Differential regulation of γ‐aminobutyric acid receptor channels by diazepam and phenobarbital. Ann Neurol. 1989;25(3):213‐220. 2471436 10.1002/ana.410250302

[10] Panas R , Brewer A III , Goldenson R . Belladonna alkaloids and phenobarbital use in the treatment of irritable bowel syndrome: a review. Clin Exp Gastroenterol Hepatol. 2018;1:102. doi:10.31531/edwiser.jcegh.1000102

[11] Ablett J . Analysis and main experience in 82 patients treated in Leeds tetanus unit. Symposium on Tetanus in Great Britain. Natinal Lending Library; 1967.

[12] National Center for Immunization and Respiratory Diseases D of BD. Tetanus, CDC; 2017. https://www.cdc.gov/tetanus/clinicians.html

[13] Hao NV , Yen LM , Davies‐Foote R , et al. The management of tetanus in adults in an intensive care unit in southern Vietnam. Wellcome OpenRes. 2021;6:6. 10.12688/wellcomeopenres.16731.1 PMC8185581 34136651

[14] Bleck TP . Pharmacology of tetanus. Clin Neuropharmacol. 1986;9(2):103‐120. 3518923 10.1097/00002826-198604000-00001

[15] Toh HC , Tiam AP . Severe tetanus in a patient with ulcerating inflammatory breast carcinoma: a case report and review of management. Acta Oncol. 1997;36(1):94‐96. 9090979 10.3109/02841869709100745

[16] Okidi R , Sambo VDC , Eyul J . Neonatal tetanus in St. Mary's hospital Lacor: a case report. Clin Case Rep. 2020;8(11):2234‐2236. 33235766 10.1002/ccr3.3091 PMC7669404

[17] Okoromah CA , Lesi AF . Diazepam for treating tetanus. Cochrane Database Syst Rev. 2004;1:CD003954. 10.1002/14651858.CD003954.pub2 PMC8759227 14974046

[18] World Health Organization . Current Recommendations for Treatment of Tetanus during Humanitarian Emergencies: WHO Technical Note. World Health Organization; 2010.

[19] Nepal G , Coghlan MA , Yadav JK , et al. Safety and efficacy of magnesium sulfate in the management of tetanus: a systematic review. Trop Med Int Health. 2021;26(10):1200‐1209. 34403179 10.1111/tmi.13667

[20] Dressnandt J , Konstanzer A , Weinzierl F , Pfab R , Klingelhöfer J . Intrathecal baclofen in tetanus: four cases and a review of reported cases. Intensive Care Med. 1997;23:896‐902. 9310810 10.1007/s001340050429

[21] Hibberd PL , Weller P , Mitty J . Tetanus‐Diphtheria Toxoid Vaccination in Adults. UpToDate; 2011.

